# Supplementary material for: HIVprotI: an integrated web based platform for prediction and design of HIV proteins inhibitors
Source: J Cheminform. 2018 Mar 9;10:12. doi: 10.1186/s13321-018-0266-y (PMC5845081; doi:10.1186/s13321-018-0266-y)
Supplement: Supplementary file 2 — Additional file 2. Source code of HIVProtI web server. [file 13321_2018_266_MOESM2_ESM.zip › HIVprotI_Source-code/hivprotI/weka-3-6-6/doc/weka/clusterers/forOPTICSAndDBScan/Utils/UpdateQueue.html]

UpdateQueue


---


|  |  |  |
| --- | --- | --- |
| **Overview** \| **Package** \| **Class** \| **Tree** \| **Deprecated** \| **Index** \| **Help** \| **Weka's home** | |  |
| **PREV CLASS**   **NEXT CLASS** | **FRAMES**    **NO FRAMES**     **All Classes** |  |
| SUMMARY: NESTED \| FIELD \| CONSTR \| METHOD | DETAIL: FIELD \| CONSTR \| METHOD |  |


---


## weka.clusterers.forOPTICSAndDBScan.Utils Class UpdateQueue

```
java.lang.Object
  weka.clusterers.forOPTICSAndDBScan.Utils.UpdateQueue
```

**All Implemented Interfaces:**: RevisionHandler

---

``` public class UpdateQueue extends java.lang.Object implements RevisionHandler ```

UpdateQueue.java

**Version:**
:   $Revision: 1.3 $

**Author:**
:   Matthias Schubert (schubert@dbs.ifi.lmu.de), Zhanna Melnikova-Albrecht (melnikov@cip.ifi.lmu.de), Rainer Holzmann (holzmann@cip.ifi.lmu.de)

---

**Constructor Summary**

`UpdateQueue()`
  
          Creates a new PriorityQueue (backed on a binary heap) with the ability to efficiently
update the priority of the stored objects in the heap.


| **Method Summary** | |
| --- | --- |
| `void` | `add(double priority, java.lang.Object o, java.lang.String objectKey)`             Adds a new Object to the queue |
| `double` | `getPriority(int index)`             Returns the priority for the object at the specified index |
| `java.lang.String` | `getRevision()`             Returns the revision string. |
| `boolean` | `hasNext()`             Tests, if the queue has some more elements left |
| `UpdateQueueElement` | `next()`             Returns the element with the lowest priority |
| `int` | `size()`             Returns the queue's size |

**Methods inherited from class java.lang.Object**

`equals, getClass, hashCode, notify, notifyAll, toString, wait, wait, wait`

**Constructor Detail**

### UpdateQueue

```
public UpdateQueue()
```

:   Creates a new PriorityQueue (backed on a binary heap) with the ability to efficiently
    update the priority of the stored objects in the heap. The ascending (!) queue is
    dynamically growing and shrinking.


**Method Detail**

### add

```
public void add(double priority,
                java.lang.Object o,
                java.lang.String objectKey)
```

:   Adds a new Object to the queue

    :   **Parameters:**: `priority` - The priority associated with the object (in this case: the reachability-distance): `objectKey` - The key for this object: `o` -

---


### getPriority

```
public double getPriority(int index)
```

:   Returns the priority for the object at the specified index

    :   **Parameters:**: `index` - the index of the object **Returns:**: priority

---


### size

```
public int size()
```

:   Returns the queue's size

    :   **Returns:**: size

---


### hasNext

```
public boolean hasNext()
```

:   Tests, if the queue has some more elements left

    :   **Returns:**: true, if there are any elements left, else false

---


### next

```
public UpdateQueueElement next()
```

:   Returns the element with the lowest priority

    :   **Returns:**: next element

---


### getRevision

```
public java.lang.String getRevision()
```

:   Returns the revision string.

    :   **Specified by:**: `getRevision` in interface `RevisionHandler`
    :   **Returns:**: the revision


---


|  |  |  |
| --- | --- | --- |
| **Overview** \| **Package** \| **Class** \| **Tree** \| **Deprecated** \| **Index** \| **Help** \| **Weka's home** | |  |
| **PREV CLASS**   **NEXT CLASS** | **FRAMES**    **NO FRAMES**     **All Classes** |  |
| SUMMARY: NESTED \| FIELD \| CONSTR \| METHOD | DETAIL: FIELD \| CONSTR \| METHOD |  |


---
